# Maximizing Without Borders: Evidence That Maximizing Transcends Decision Domains

**DOI:** 10.3389/fpsyg.2018.02664

**Published:** 2019-01-15

**Authors:** Michail D. Kokkoris

**Affiliations:** ^1^Department of Marketing, WU Vienna University of Economics and Business, Vienna, Austria

**Keywords:** maximizing, decision making, decision domains, consumer goods, experiences

## Abstract

Do maximizers maximize across decision domains? An assumption underlying the literature on maximizing is that the tendency to strive to make the best choice spans domains. The current research provides a direct test of this assumption by examining the association between trait maximizing and domain-specific maximizing, consisting of maximizing measures in a wide range of decisions (consumer goods, services and experiences, and life decisions). Study 1 tested this association at two different time points in order to minimize common method bias. Study 2 was a high-powered pre-registered cross-sectional replication. Results of both studies showed that trait maximizing was associated with higher maximizing tendencies across all three decision domains. However, in line with prior research suggesting that people generally maximize less in experiential than in material domains, trait maximizing was associated with maximizing in services and experiences significantly less than with maximizing in consumer goods or in life decisions. These results provide empirical support for a central tenet of maximizing theory and suggest useful directions for future research.

## Introduction

The construct of maximizing describes individual differences in the tendency to strive to make the best choice (for reviews, see Cheek and Schwartz, 2016; Misuraca and Fasolo, 2018). Implicit in the research on maximizing is the assumption that this tendency is expressed across decision domains (Schwartz et al., 2002; Diab et al., 2008; Misuraca et al., 2015). Although this assumption seems to be a central tenet of the maximizing theory, not much research has put this explicitly into test.

Initial supportive evidence has been provided by Diab et al. (2008) and Misuraca et al. (2015), but only in a limited number of decision domains (car purchase, shopping for clothes, job search, housing search, graduate school offers; consumer and professional/career domain, respectively). Evidence across many more decision domains (e.g., clothes, cars, movies, sex life, job offers, friends, school grades, or yearly income) has been provided by Weaver et al. (2015). However, a limitation of this study is that it used the Maximization Scale (Schwartz et al., 2002), which has been criticized for not adequately measuring maximizing (Diab et al., 2008; Lai, 2010; Rim et al., 2011; Turner et al., 2012; Weinhardt et al., 2012; Richardson et al., 2014; Dalal et al., 2015; Misuraca et al., 2015). For example, this scale might be confounding maximizing (having high standards when choosing) with decision difficulty (experiencing difficulty in choosing) and alternative search (searching extensively before choosing), which may be correlates of the maximizing construct but not parts of its definition. Other weaknesses of the Maximization Scale include poor psychometric properties and outdated items (e.g., about video rental). These limitations have led the authors of this scale to later revise it (Nenkov et al., 2008) and more recently even recommend other scales as better alternatives (Cheek and Schwartz, 2016).

The current study constitutes the first systematic attempt to directly test the assumption that maximizers maximize across a wide range of decision domains by overcoming the shortcomings of prior studies that either tested this assumption in a limited number of domains or used problematic measures of maximizing. In line with prior theorizing, maximizing was conceptualized as a global decision-making tendency that is reflected in various decision tasks. The present article examined whether trait maximizing is associated with maximizing in decisions concerning consumer goods, decisions concerning experiences and services, and important life decisions. Decisions related to consumer goods have been extensively studied in the maximizing literature (e.g., Diab et al., 2008; Weaver et al., 2015; Kokkoris, 2018). Decisions related to experiences and services have been studied in the maximizing literature to a lesser degree. It has been suggested that people are generally less likely to maximize in the experiential domain, mainly because decision outcomes in this domain are harder to compare and thus one can never know what the best choice really is (Carter and Gilovich, 2010). For example, whereas one can strive to choose the best smartphone (consumer good) based on some objective criteria, this is harder to do for a holiday (experience). Finally, life decisions have also been examined in the context of maximizing. A general maximizing tendency has been associated with maximizing in romantic relationships (Mikkelson and Pauley, 2013), friendships (Newman et al., 2018), or academic and professional decisions (Iyengar et al., 2006; Dahling and Thompson, 2013; Leach and Patall, 2013). However, conclusions drawn from these studies are again limited due to the use of the Maximization Scale.

## Study 1

Study 1 examined the associations (assessed in two separate time points to minimize common method bias) between trait maximizing and maximizing tendencies across a variety of decision domains.

### Methods

#### Participants

One-hundred thirty-two United States residents recruited on Prolific Academic participated in the first survey. All participants who took the first survey were contacted 3 days later and were asked to take part in a follow-up survey. Of these, 78 participants (59%) took part in it. The analysis sample therefore comprised 78 participants (53 men, 25 women, age 18–74, *M* = 32.72, *SD* = 13.71). Participants in the final analysis sample (*M* = 4.74, *SD* = 1.04; *n* = 78) did not differ significantly in maximizing from those who dropped out after the first survey (*M* = 4.86, *SD* = 0.84; *n* = 52), *t*(130) = 0.68, *p* = 0.50. An *a priori* power analysis with G^∗^Power3 (Faul et al., 2007) revealed that the sample size collected can reliably detect medium effect sizes of ρ = 0.28 (one-tailed) with an alpha level of 0.05 and power of 0.80.

#### Procedure

In the first survey, participants filled out the Maximizing Tendency Scale (α = 0.90; Diab et al., 2008), which assesses only high standards and has been recommended as the most suitable measure of the maximizing goal among the currently available alternatives (Cheek and Schwartz, 2016). The scale includes items like “No matter what it takes, I always try to choose the best thing” on a 7-point scale (1 – *strongly disagree*; 7 – *strongly agree*). In the second survey, participants were presented with a list of 29 decision domains: consumer goods (bottled water; food; detergent; clothes; shoes; sunglasses; perfume; furniture; smartphone; laptop; car); experiences and services (gym; film; book; concert; TV series; restaurant; meal in a restaurant; café/bar; drink in a bar; hotel room; holiday destination); and life decisions (area of residence; apartment; job; employer; studies; friends; partner). For each domain, participants had to indicate on a 6-point scale to what extent they want to make the best choice in the respective domain (6) or it is enough for them to choose an option that is satisfactory and good enough (1). The presentation order of the domains was randomized for every participant. See exact wording of all items in the Appendix in Supplementary Table 3.

### Results

**FIGURE 1:**
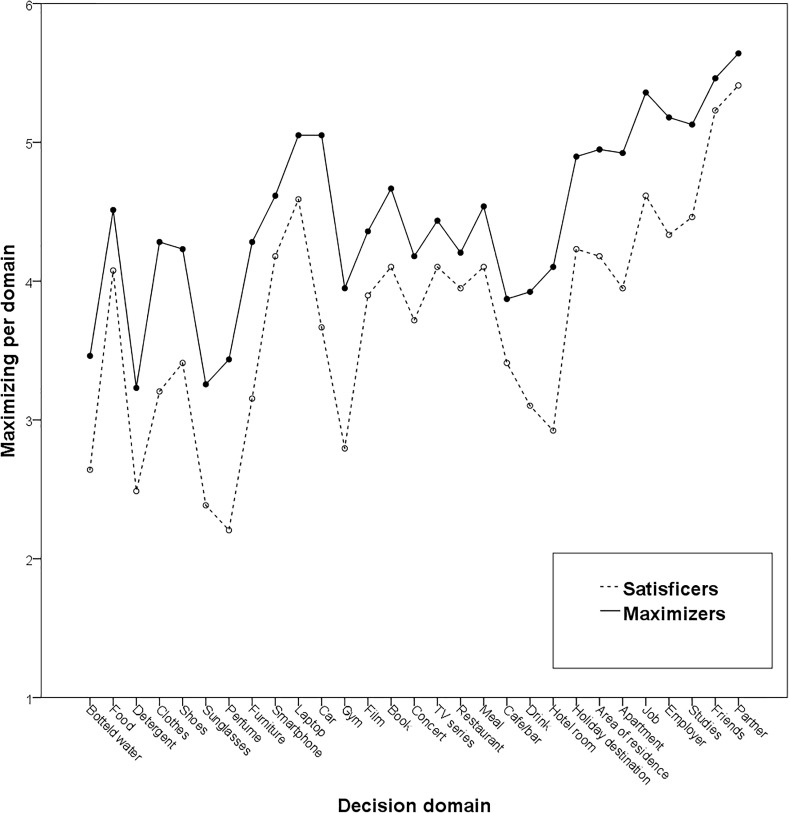
Satisficers’ and maximizers’ maximizing tendencies per decision domain in Study 1.

Results showed significant positive correlations of trait maximizing with maximizing in 16 decision domains (Supplementary Table 1). For ease of visualization, Figure 1 presents maximizers’ and satisficers’ (based on a median split) maximizing scores on each decision domain. As the maximizing scores per domain displayed good reliability (α = 0.92), a composite score of aggregate domain-specific maximizing was computed. Trait maximizing correlated positively with this aggregate domain-specific maximizing score, *r* = 0.42, *p* < 0.001. Moreover, separate scores for the three decision domains were also computed: consumer goods (α = 0.88), experiences and services (α = 0.85), and life decisions (α = 0.81). Trait maximizing correlated positively with all three: *r* = 0.38, *p* = 0.001; *r* = 0.27, *p* = 0.015; and *r* = 0.45, *p* < 0.001, respectively.

### Discussion

Study 1 provides preliminary evidence that maximizers maximize across decision domains, from consumer choices to consequential life decisions. Importantly, the two time-points design helps reduce concerns about measurement bias and suggests that this association most likely represents a genuine relationship. However, as the power analysis implies, the current study might be underpowered to detect small to medium effect sizes, which might be critical for the smaller effect of the three, namely the association between general maximizing and domain-specific maximizing for experiences and services. Study 2 addressed this issue.

## Study 2

Study 2 was a pre-registered replication of Study 1 with a larger sample capable of detecting smaller effect sizes. A positive association between trait maximizing and domain-specific maximizing was hypothesized. To minimize common method bias in cross-sectional designs, the order of the two measures was counterbalanced, different answering scales were used, and a filler task was interspersed. Hypotheses, measures, data collection, and analyses were preregistered on the Open Science Framework website^1^.

### Methods

#### Participants

Two-hundred ninety-seven Austrian residents recruited via the mailing list of a large Austrian university participated in an online survey. The final sample comprised 227 participants (84 men, 143 women, age 18–55, *M* = 24.42, *SD* = 6.66) who completed the entire survey. An *a priori* power analysis with G^∗^Power3 (Faul et al., 2007) revealed that the sample size collected can reliably detect small to medium effect sizes of ρ = 0.17 (one-tailed) with an alpha level of 0.05 and power of 0.80.

#### Procedure

Participants completed the same measures of trait maximizing (α = 0.78) and domain-specific maximizing (α = 0.87) as in Study 1. The order of the two measures was counterbalanced across participants. Between the two measures, another study on an unrelated topic was interspersed as a filler task.

### Results

**FIGURE 2:**
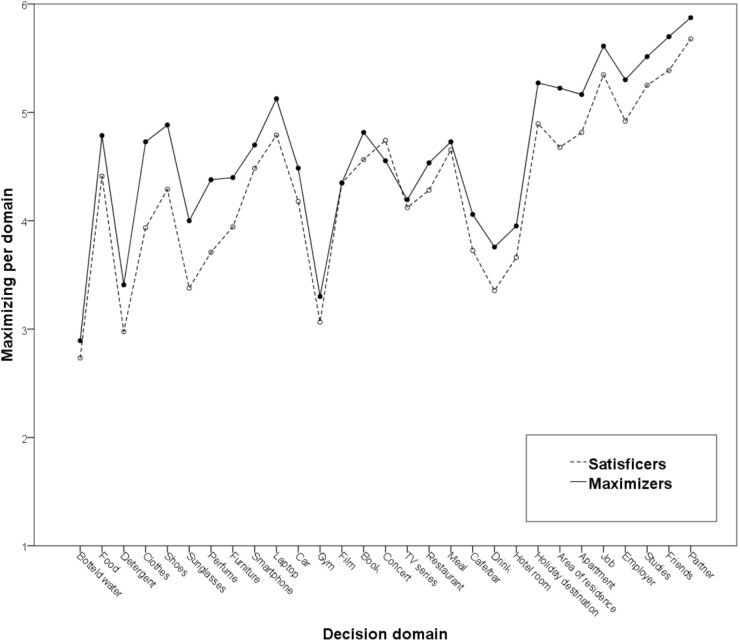
Satisficers’ and maximizers’ maximizing tendencies per decision domain in Study 2.

Results showed significant positive correlations of trait maximizing with maximizing in 22 decision domains (Supplementary Table 2). Using again a median split for visualization purposes, Figure 2 presents maximizers’ and satisficers’ maximizing scores on each domain. Analyses based on an aggregate domain-specific maximizing score showed that trait maximizing was positively correlated with domain-specific maximizing, *r* = 0.41, *p* < 0.001. Moreover, as in Study 1, separate scores for maximizing in consumer goods (α = 0.80), experiences and services (α = 0.78), and life decisions (α = 0.77) were computed. Trait maximizing was positively correlated with all three: *r* = 0.38, *p* < 0.001; *r* = 0.25, *p* < 0.001; and *r* = 0.40, *p* < 0.001, respectively.

### Discussion

Study 2 provides additional evidence that maximizing transcends decision domains. Additionally, with a sample size large enough to detect small to medium effect sizes, these results lend more confidence that the positive association between trait maximizing and domain-specific maximizing concerns all three decision domains examined (consumer goods, experiences and services, and life decisions).

## Internal Meta-Analysis and Follow-Up Analyses

An internal meta-analysis of both studies with a random-effects model applying the Hedges and Vevea (1998) method showed that the mean effect size for the correlation between trait maximizing and maximizing for consumer goods was 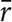 = 0.38, 95% CI = [0.28, 0.47], *z* = 6.92, *p* < 0.001; for experiences and services it was 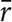 = 0.26, 95% CI = [0.15, 0.36], *z* = 4.51, *p* < 0.001; and for life decisions it was 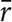 = 0.41, 95% CI = [0.32, 0.50], *z* = 7.59, *p* < 0.001. Therefore, maximizing was positively correlated with maximizing in all three domains, consumer goods, experiences and services, and life decisions. However, follow-up analyses with pooled data of both studies showed that maximizers maximize in services and experiences significantly less than in consumer goods, Steiger’s *z* = 2.22, *p* = 0.026, or in life decisions, Steiger’s *z* = 2.42, *p* = 0.015, whereas the extent to which they maximize does not differ between life decisions and consumer goods, Steiger’s *z* = -0.33, *p* = 0.608 (Steiger, 1980; Lee and Preacher, 2013).

## General Discussion

These findings make a significant contribution to the maximizing literature as they provide the first explicit test of the long-held assumption that maximizing is domain-spanning. The current research goes beyond prior research in the following ways. First, although some previous studies have provided evidence that maximizing is expressed in various domains (Iyengar et al., 2006; Dahling and Thompson, 2013; Leach and Patall, 2013; Mikkelson and Pauley, 2013; Weaver et al., 2015; Newman et al., 2018), all of these studies used a rather inadequate measure of maximizing (Schwartz et al., 2002). The present research, using a much more appropriate measure of maximizing (Diab et al., 2008), was able to provide more valid evidence in support of this association. Second, even the studies that used more sound measures of maximizing often provided evidence in only a few domains (Diab et al., 2008; Misuraca et al., 2015). The current research encompasses a much wider range of domains in a more systematic way. Third, unlike prior research that relied mostly on cross-sectional designs, the current research used a two-stage design that assessed the main variables in different time points (Study 1). Another contribution of this article is that it extends research on people’s tendency to maximize less in experiential than material purchases (Carter and Gilovich, 2010) by showing that maximizers follow this pattern, too. Future research could employ behavioral tasks as a more direct measure of domain-specific maximizing beyond the self-report measures used here, as well as expand decision domains to medical or financial decision making.

## Ethics Statement

Full review and approval by an ethics committee was not required according to the local and national guidelines. All subjects gave written informed consent in accordance with the Declaration of Helsinki.

## Author Contributions

The author confirms being the sole contributor of this work and has approved it for publication.

## Conflict of Interest Statement

The author declares that the research was conducted in the absence of any commercial or financial relationships that could be construed as a potential conflict of interest.

## Footnotes

1 https://osf.io/dnu92/?view_only=fdaa368a97d440d0bb9b9bdf40a20daf
