# Supplementary material for: Maximizing Without Borders: Evidence That Maximizing Transcends Decision Domains
Source: Front Psychol. 2019 Jan 15;9:2664. doi: 10.3389/fpsyg.2018.02664 (PMC6340960; doi:10.3389/fpsyg.2018.02664)
Supplement: Supplementary file 1 [file Table_1.DOCX]

Table 1a

Descriptive Statistics and Inter-Correlations of Variables in Study 1 (part I)

|  | **1** | **2** | **3** | **4** | **5** | **6** | **7** | **8** | **9** | **10** | **11** | **12** | **13** | **14** | **15** |
| --- | --- | --- | --- | --- | --- | --- | --- | --- | --- | --- | --- | --- | --- | --- | --- |
| **1. Trait maximizing** | - |  |  |  |  |  |  |  |  |  |  |  |  |  |  |
| **2. Domain-specific maxim.** | .416^**^ | - |  |  |  |  |  |  |  |  |  |  |  |  |  |
| **3. Bottled water** | .158 | .473^**^ | - |  |  |  |  |  |  |  |  |  |  |  |  |
| **4. Food** | .066 | .581^**^ | .447^**^ | - |  |  |  |  |  |  |  |  |  |  |  |
| **5. Detergent** | .204 | .603^**^ | .404^**^ | .347^**^ | - |  |  |  |  |  |  |  |  |  |  |
| **6. Clothes** | .272^*^ | .689^**^ | .350^**^ | .394^**^ | .433^**^ | - |  |  |  |  |  |  |  |  |  |
| **7. Shoes** | .232^*^ | .667^**^ | .320^**^ | .432^**^ | .405^**^ | .731^**^ | - |  |  |  |  |  |  |  |  |
| **8. Perfume** | .293^**^ | .722^**^ | .391^**^ | .366^**^ | .515^**^ | .511^**^ | .555^**^ | - |  |  |  |  |  |  |  |
| **9. Sunglasses** | .266^*^ | .664^**^ | .303^**^ | .222 | .460^**^ | .595^**^ | .490^**^ | .542^**^ | - |  |  |  |  |  |  |
| **10. Furniture** | .328^**^ | .702^**^ | .316^**^ | .365^**^ | .538^**^ | .671^**^ | .578^**^ | .346^**^ | .561^**^ | - |  |  |  |  |  |
| **11. Smartphone** | .249^*^ | .577^**^ | .071 | .296^**^ | .355^**^ | .479^**^ | .519^**^ | .296^**^ | .415^**^ | .498^**^ | - |  |  |  |  |
| **12. Laptop** | .226^*^ | .612^**^ | .143 | .469^**^ | .344^**^ | .343^**^ | .499^**^ | .302^**^ | .301^**^ | .446^**^ | .667^**^ | - |  |  |  |
| **13. Car** | .516^**^ | .563^**^ | .170 | .111 | .329^**^ | .356^**^ | .463^**^ | .449^**^ | .314^**^ | .469^**^ | .547^**^ | .485^**^ | - |  |  |
| **14. Gym** | .311^**^ | .630^**^ | .272^*^ | .441^**^ | .404^**^ | .367^**^ | .299^**^ | .342^**^ | .389^**^ | .432^**^ | .387^**^ | .398^**^ | .294^**^ | - |  |
| **15. Film** | .087 | .469^**^ | .194 | .143 | .191 | .157 | .131 | .339^**^ | .234^*^ | .221 | .115 | .093 | .104 | .203 | - |
| **16. Book** | .259^*^ | .424^**^ | -.022 | .092 | .166 | .161 | .139 | .301^**^ | .355^**^ | .302^**^ | .258^*^ | .197 | .301^**^ | .238^*^ | .411^**^ |
| **17. Concert** | .109 | .504^**^ | .082 | .014 | .292^**^ | .335^**^ | .257^*^ | .388^**^ | .484^**^ | .418^**^ | .385^**^ | .191 | .215 | .311^**^ | .471^**^ |
| **18. TV series** | .081 | .442^**^ | .043 | .179 | .085 | .170 | .174 | .354^**^ | .266^*^ | .087 | .249^*^ | .190 | .088 | .185 | .629^**^ |
| **19. Restaurant** | .057 | .659^**^ | .263^*^ | .378^**^ | .322^**^ | .468^**^ | .422^**^ | .436^**^ | .482^**^ | .437^**^ | .314^**^ | .334^**^ | .227^*^ | .217 | .428^**^ |
| **20. Meal in a restaurant** | .103 | .619^**^ | .395^**^ | .521^**^ | .447^**^ | .384^**^ | .338^**^ | .406^**^ | .315^**^ | .429^**^ | .218 | .419^**^ | .043 | .330^**^ | .260^*^ |
| **21. Café/bar** | .172 | .612^**^ | .274^*^ | .367^**^ | .223^*^ | .462^**^ | .424^**^ | .515^**^ | .462^**^ | .390^**^ | .241^*^ | .170 | .157 | .163 | .549^**^ |
| **22. Drink in a café/bar** | .220 | .660^**^ | .340^**^ | .423^**^ | .347^**^ | .376^**^ | .293^**^ | .470^**^ | .487^**^ | .365^**^ | .287^*^ | .263^*^ | .133 | .489^**^ | .380^**^ |
| **23. Hotel room** | .333^**^ | .547^**^ | .422^**^ | .329^**^ | .348^**^ | .258^*^ | .313^**^ | .413^**^ | .288^*^ | .341^**^ | .228^*^ | .335^**^ | .355^**^ | .454^**^ | .058 |
| **24. Holiday destination** | .159 | .602^**^ | .101 | .291^**^ | .295^**^ | .433^**^ | .353^**^ | .396^**^ | .484^**^ | .449^**^ | .206 | .230^*^ | .180 | .402^**^ | .382^**^ |
| **25. Area of residence** | .308^**^ | .556^**^ | .299^**^ | .507^**^ | .251^*^ | .416^**^ | .334^**^ | .395^**^ | .246^*^ | .255^*^ | .142 | .332^**^ | .131 | .306^**^ | .057 |
| **26. Apartment** | .291^**^ | .623^**^ | .341^**^ | .297^**^ | .379^**^ | .359^**^ | .360^**^ | .421^**^ | .327^**^ | .378^**^ | .242^*^ | .362^**^ | .434^**^ | .472^**^ | .037 |
| **27. Job** | .342^**^ | .561^**^ | .290^*^ | .467^**^ | .160 | .267^*^ | .270^*^ | .279^*^ | .096 | .253^*^ | .169 | .369^**^ | .350^**^ | .336^**^ | .207 |
| **28. Employer** | .380^**^ | .633^**^ | .246^*^ | .434^**^ | .279^*^ | .242^*^ | .302^**^ | .433^**^ | .214 | .254^*^ | .240^*^ | .417^**^ | .416^**^ | .442^**^ | .301^**^ |
| **29. Studies** | .350^**^ | .336^**^ | -.072 | .093 | .172 | .123 | .109 | .127 | .047 | .216 | .194 | .358^**^ | .484^**^ | .370^**^ | .094 |
| **30. Friends** | .214 | .358^**^ | .153 | .124 | .032 | .191 | .227^*^ | .176 | .080 | .156 | .147 | .263^*^ | .218 | .130 | .185 |
| **31. Partner** | .207 | .220 | -.123 | .037 | .032 | -.005 | -.008 | .051 | .066 | .115 | .121 | .237^*^ | .203 | .097 | .089 |
| *M* | 4.74 | 4.10 | 3.05 | 4.29 | 2.86 | 3.74 | 3.82 | 2.82 | 2.82 | 3.72 | 4.40 | 4.82 | 4.36 | 3.37 | 4.13 |
| *SD* | 1.04 | 0.76 | 1.72 | 1.28 | 1.41 | 1.49 | 1.32 | 1.70 | 1.56 | 1.49 | 1.44 | 1.26 | 1.62 | 1.46 | 1.42 |

Table 1b

Descriptive Statistics and Inter-Correlations of Variables in Study 1 (part II)

|  | **16** | **17** | **18** | **19** | **20** | **21** | **22** | **23** | **24** | **25** | **26** | **27** | **28** | **29** | **30** | **31** |
| --- | --- | --- | --- | --- | --- | --- | --- | --- | --- | --- | --- | --- | --- | --- | --- | --- |
| **16. Book** | - |  |  |  |  |  |  |  |  |  |  |  |  |  |  |  |
| **17. Concert** | .376^**^ | - |  |  |  |  |  |  |  |  |  |  |  |  |  |  |
| **18. TV series** | .459^**^ | .333^**^ | - |  |  |  |  |  |  |  |  |  |  |  |  |  |
| **19. Restaurant** | .318^**^ | .274^*^ | .390^**^ | - |  |  |  |  |  |  |  |  |  |  |  |  |
| **20. Meal in a restaurant** | .107 | .279^*^ | .250^*^ | .616^**^ | - |  |  |  |  |  |  |  |  |  |  |  |
| **21. Café/bar** | .361^**^ | .407^**^ | .352^**^ | .560^**^ | .479^**^ | - |  |  |  |  |  |  |  |  |  |  |
| **22. Drink in a café/bar** | .183 | .490^**^ | .314^**^ | .407^**^ | .555^**^ | .553^**^ | - |  |  |  |  |  |  |  |  |  |
| **23. Hotel room** | -.018 | .024 | .081 | .291^**^ | .417^**^ | .156 | .351^**^ | - |  |  |  |  |  |  |  |  |
| **24. Holiday destination** | .245^*^ | .478^**^ | .297^**^ | .438^**^ | .488^**^ | .444^**^ | .579^**^ | .271^*^ | - |  |  |  |  |  |  |  |
| **25. Area of residence** | -.008 | .036 | .190 | .414^**^ | .450^**^ | .248^*^ | .329^**^ | .493^**^ | .265^*^ | - |  |  |  |  |  |  |
| **26. Apartment** | -.008 | .203 | .204 | .329^**^ | .381^**^ | .139 | .382^**^ | .642^**^ | .316^**^ | .635^**^ | - |  |  |  |  |  |
| **27. Job** | .127 | -.048 | .226^*^ | .401^**^ | .273^*^ | .301^**^ | .240^*^ | .361^**^ | .212 | .629^**^ | .513^**^ | - |  |  |  |  |
| **28. Employer** | .303^**^ | .113 | .265^*^ | .345^**^ | .236^*^ | .357^**^ | .333^**^ | .347^**^ | .242^*^ | .553^**^ | .432^**^ | .790^**^ | - |  |  |  |
| **29. Studies** | .308^**^ | .033 | -.012 | .167 | .083 | .058 | .102 | -.006 | .244^*^ | .035 | .116 | .389^**^ | .441^**^ | - |  |  |
| **30. Friends** | .150 | .014 | .155 | .194 | .132 | .195 | .136 | .218 | .010 | .306^**^ | .365^**^ | .401^**^ | .330^**^ | .206 | - |  |
| **31. Partner** | .188 | .042 | .069 | .075 | -.045 | -.016 | .057 | .151 | .037 | .122 | .152 | .291^**^ | .382^**^ | .285^*^ | .629^**^ | - |
| *M* | 4.38 | 3.95 | 4.27 | 4.08 | 4.32 | 3.64 | 3.51 | 3.51 | 4.56 | 4.56 | 4.44 | 4.99 | 4.76 | 4.79 | 5.35 | 5.53 |
| *SD* | 1.23 | 1.59 | 1.40 | 1.05 | 1.12 | 1.28 | 1.42 | 1.39 | 1.25 | 1.18 | 1.40 | 1.20 | 1.23 | 1.27 | 0.82 | 0.80 |

Note. ** *p* < .01 (2-tailed); * *p* < .05 (2-tailed)
