# Supplementary material for: Maximizing Without Borders: Evidence That Maximizing Transcends Decision Domains
Source: Front Psychol. 2019 Jan 15;9:2664. doi: 10.3389/fpsyg.2018.02664 (PMC6340960; doi:10.3389/fpsyg.2018.02664)
Supplement: Supplementary file 2 [file Table_2.DOCX]

Table 2a

Descriptive Statistics and Inter-Correlations of Variables in Study 2 (part I)

|  | **1** | **2** | **3** | **4** | | **5** | **6** | **7** | **8** | **9** | **10** | **11** | **12** | **13** | **14** | **15** |
| --- | --- | --- | --- | --- | --- | --- | --- | --- | --- | --- | --- | --- | --- | --- | --- | --- |
| **1. Trait maximizing** | - |  |  |  | |  |  |  |  |  |  |  |  |  |  |  |
| **2. Domain-specific maxim.** | .413^**^ | - |  |  | |  |  |  |  |  |  |  |  |  |  |  |
| **3. Bottled water** | .105 | .412^**^ | - |  | |  |  |  |  |  |  |  |  |  |  |  |
| **4. Food** | .229^**^ | .499^**^ | .395^**^ | - | |  |  |  |  |  |  |  |  |  |  |  |
| **5. Detergent** | .189^**^ | .448^**^ | .285^**^ | .306^**^ | | - |  |  |  |  |  |  |  |  |  |  |
| **6. Clothes** | .299^**^ | .590^**^ | .149^*^ | .259^**^ | | .367^**^ | - |  |  |  |  |  |  |  |  |  |
| **7. Shoes** | .362^**^ | .602^**^ | .092 | .250^**^ | | .300^**^ | .577^**^ | - |  |  |  |  |  |  |  |  |
| **8. Perfume** | .286^**^ | .497^**^ | .103 | .135^*^ | | .170^*^ | .420^**^ | .442^**^ | - |  |  |  |  |  |  |  |
| **9. Sunglasses** | .308^**^ | .521^**^ | .254^**^ | .224^**^ | | .254^**^ | .376^**^ | .476^**^ | .255^**^ | - |  |  |  |  |  |  |
| **10. Furniture** | .227^**^ | .542^**^ | .120 | .195^**^ | | .276^**^ | .407^**^ | .465^**^ | .264^**^ | .294^**^ | - |  |  |  |  |  |
| **11. Smartphone** | .118 | .437^**^ | -.004 | .088 | | .145^*^ | .253^**^ | .265^**^ | .230^**^ | .243^**^ | .257^**^ | - |  |  |  |  |
| **12. Laptop** | .199^**^ | .543^**^ | .076 | .229^**^ | | .191^**^ | .280^**^ | .375^**^ | .328^**^ | .306^**^ | .399^**^ | .574^**^ | - |  |  |  |
| **13. Car** | .105 | .416^**^ | .054 | .054 | | .188^**^ | .280^**^ | .266^**^ | .209^**^ | .239^**^ | .377^**^ | .473^**^ | .440^**^ | - |  |  |
| **14. Gym** | .182^**^ | .480^**^ | .311^*^ | .288^**^ | | .262^**^ | .158^*^ | .199^**^ | .144^*^ | .373^**^ | .179^**^ | .268^**^ | .291^**^ | .330^**^ | - |  |
| **15. Film** | .104 | .381^**^ | .085 | .087 | | -.001 | .064 | .176^**^ | .145^*^ | .181^**^ | .041 | .044 | .071 | .017 | .185^**^ | - |
| **16. Book** | .176^**^ | .256^**^ | .055 | .134^*^ | | .031 | .031 | .180^**^ | .107 | .026 | .093 | -.037 | .013 | -.075 | -.005 | .387^**^ |
| **17. Concert** | -.041 | .404^**^ | .101 | .127 | | .111 | .095 | .093 | .168^*^ | .059 | .097 | .146^*^ | .147^*^ | .165^*^ | .108 | .293^**^ |
| **18. TV series** | .080 | .356^**^ | -.009 | .039 | | -.004 | .079 | .157^*^ | .094 | .081 | .006 | .087 | .074 | .023 | .042 | .502^**^ |
| **19. Restaurant** | .155^*^ | .591^**^ | .257^**^ | .415^**^ | | .253^**^ | .273^**^ | .271^**^ | .131^*^ | .266^**^ | .250^**^ | .229^**^ | .273^**^ | .153^*^ | .268^**^ | .191^**^ |
| **20. Meal in a restaurant** | .116 | .542^**^ | .275^**^ | .385^**^ | | .202^**^ | .215^**^ | .232^**^ | .210^**^ | .182^**^ | .180^**^ | .133^*^ | .155^*^ | .065 | .221^**^ | .208^**^ |
| **21. Café/bar** | .166^*^ | .555^**^ | .349^**^ | .309^**^ | | .162^*^ | .298^**^ | .229^**^ | .168^*^ | .181^**^ | .145^*^ | .085 | .197^**^ | .088 | .282^**^ | .208^**^ |
| **22. Drink in a café/bar** | .243^**^ | .561^**^ | .161^*^ | .213^**^ | | .266^**^ | .259^**^ | .339^**^ | .239^**^ | .286^**^ | .268^**^ | .126 | .172^**^ | .029 | .226^**^ | .351^**^ |
| **23. Hotel room** | .138^*^ | .508^**^ | .197^**^ | .207^**^ | | .215^**^ | .248^**^ | .182^**^ | .121 | .221^**^ | .258^**^ | .219^**^ | .242^**^ | .235^**^ | .288^**^ | .086 |
| **24. Holiday destination** | .250^**^ | .589^**^ | .227^**^ | .236^**^ | | .194^**^ | .350^**^ | .355^**^ | .258^**^ | .187^**^ | .321^**^ | .124 | .239^**^ | .175^**^ | .196^**^ | .139^*^ |
| **25. Area of residence** | .320^**^ | .478^**^ | .176^**^ | .315^**^ | | .060 | .201^**^ | .144^*^ | .170^*^ | .086 | .277^**^ | .036 | .182^**^ | .094 | .139^*^ | .132^*^ |
| **26. Apartment** | .219^**^ | .504^**^ | .169^*^ | .151^*^ | | .088 | .329^**^ | .221^**^ | .195^**^ | .155^*^ | .394^**^ | .180^**^ | .230^**^ | .208^**^ | .205^**^ | .030 |
| **27. Job** | .308^**^ | .389^**^ | .061 | .135^*^ | | .029 | .145^*^ | .173^**^ | .142^*^ | .113 | .194^**^ | .131^*^ | .136^*^ | .070 | .003 | .129 |
| **28. Employer** | .226^**^ | .381^**^ | .030 | .104 | | .188^**^ | .251^**^ | .078 | .190^**^ | .118 | .053 | .106 | .126 | .064 | .019 | .139^*^ |
| **29. Studies** | .293^**^ | .409^**^ | .063 | .142^*^ | | .120 | .264^**^ | .259^**^ | .274^**^ | .142^*^ | .209^**^ | .168^*^ | .244^**^ | -.014 | .046 | .063 |
| **30. Friends** | .232^**^ | .364^**^ | .173^**^ | .252^**^ | | .047 | .161^*^ | .104 | .230^**^ | .087 | .076 | .085 | .180^**^ | -.044 | -.022 | .041 |
| **31. Partner** | .237^**^ | .321^**^ | .046 | .096 | .079 | | .157^*^ | .218^**^ | .110 | .030 | .171^**^ | .124 | .116 | .012 | -.013 | .148* |
| *M* | 4.61 | 4.43 | 2.81 | 4.58 | | 3.17 | 4.30 | 4.56 | 4.01 | 3.66 | 4.15 | 4.58 | 4.94 | 4.32 | 3.17 | 4.35 |
| *SD* | 0.87 | 0.61 | 1.74 | 1.25 | | 1.46 | 1.37 | 1.23 | 1.58 | 1.58 | 1.42 | 1.41 | 1.23 | 1.64 | 1.55 | 1.30 |
|  |  |  |  |  | |  |  |  |  |  |  |  |  |  |  |  |

Table 2b

Descriptive Statistics and Inter-Correlations of Variables in Study 2 (part II)

|  | **16** | **17** | **18** | **19** | **20** | **21** | **22** | **23** | **24** | **25** | **26** | **27** | **28** | **29** | **30** | **31** |
| --- | --- | --- | --- | --- | --- | --- | --- | --- | --- | --- | --- | --- | --- | --- | --- | --- |
| **16. Book** | - |  |  |  |  |  |  |  |  |  |  |  |  |  |  |  |
| **17. Concert** | .219^**^ | - |  |  |  |  |  |  |  |  |  |  |  |  |  |  |
| **18. TV series** | .225^**^ | .274^**^ | - |  |  |  |  |  |  |  |  |  |  |  |  |  |
| **19. Restaurant** | .101 | .195^**^ | .181^**^ | - |  |  |  |  |  |  |  |  |  |  |  |  |
| **20. Meal in a restaurant** | .179^**^ | .221^**^ | .160^*^ | .589^**^ | - |  |  |  |  |  |  |  |  |  |  |  |
| **21. Café/bar** | .178^**^ | .271^**^ | .138^*^ | .510^**^ | .431^**^ | - |  |  |  |  |  |  |  |  |  |  |
| **22. Drink in a café/bar** | .195^**^ | .239^**^ | .254^**^ | .432^**^ | .438^**^ | .490^**^ | - |  |  |  |  |  |  |  |  |  |
| **23. Hotel room** | -.068 | .150^*^ | .119 | .425^**^ | .306^**^ | .370^**^ | .207^**^ | - |  |  |  |  |  |  |  |  |
| **24. Holiday destination** | .069 | .261^**^ | .249^**^ | .380^**^ | .356^**^ | .321^**^ | .281^**^ | .257^**^ | - |  |  |  |  |  |  |  |
| **25. Area of residence** | .081 | .141* | .204^**^ | .143^*^ | .220^**^ | .152^*^ | .245^**^ | .190^**^ | .420^**^ | - |  |  |  |  |  |  |
| **26. Apartment** | -.020 | .110 | .103 | .176^**^ | .131^*^ | .255^**^ | .159^*^ | .458^**^ | .345^**^ | .469^**^ | - |  |  |  |  |  |
| **27. Job** | .178^**^ | .212^**^ | .180^**^ | .165^*^ | .114 | .048 | .134^*^ | .173^**^ | .290^**^ | .307^**^ | .278^**^ | - |  |  |  |  |
| **28. Employer** | -.016 | .161^*^ | .233^**^ | .072 | .098 | .126 | .059 | .185^**^ | .226^**^ | .407^**^ | .390^**^ | .434^**^ | - |  |  |  |
| **29. Studies** | .074 | .094 | .183^**^ | .102 | .187^**^ | .097 | .135^*^ | .075 | .298^**^ | .330^**^ | .299^**^ | .352^**^ | .357^**^ | - |  |  |
| **30. Friends** | .049 | .070 | .143^*^ | .093 | .215^**^ | .261^**^ | .144^*^ | .067 | .339^**^ | .387^**^ | .195^**^ | .276^**^ | .340^**^ | .410^**^ | - |  |
| **31. Partner** | .243^**^ | .129 | .177^**^ | .164^*^ | .059 | .033 | .175^**^ | .045 | .207^**^ | .252^**^ | .199^**^ | .484^**^ | .157^*^ | .268^**^ | .323^**^ | - |
| *M* | 4.68 | 4.66 | 4.15 | 4.40 | 4.69 | 3.88 | 3.54 | 3.79 | 5.10 | 4.93 | 4.97 | 5.47 | 5.10 | 5.37 | 5.53 | 5.80 |
| *SD* | 1.33 | 1.41 | 1.42 | 1.24 | 1.19 | 1.34 | 1.45 | 1.52 | 1.10 | 1.21 | 1.14 | 0.82 | 1.12 | 0.90 | 0.86 | 0.65 |

Note. ** *p* < .01 (2-tailed); * *p* < .05 (2-tailed)
