# Supplementary material for: Maximizing Without Borders: Evidence That Maximizing Transcends Decision Domains
Source: Front Psychol. 2019 Jan 15;9:2664. doi: 10.3389/fpsyg.2018.02664 (PMC6340960; doi:10.3389/fpsyg.2018.02664)
Supplement: Supplementary file 3 [file Table_3.DOCX]

**APPENDIX**

Maximizing without Borders: Evidence that Maximizing Transcends Decision Domains

**General maximizing**

Please indicate to what extent you agree or disagree with the following statements. (1 = strongly disagree; 7 = strongly agree)

- No matter what it takes, I always try to choose the best thing.
- I don’t like having to settle for “good enough”.
- I am a maximizer.
- No matter what I do, I have the highest standards for myself.
- I will wait for the best option, no matter how long it takes.
- I never settle for second best.
- I am uncomfortable making decisions before I know all of my options.
- Whenever I’m faced with a choice, I try to imagine what all the other possibilities are, even ones that aren’t present at the moment.
- I never settle.

**Domain-specific maximizing**

The following questions concern choices in various domains.

Please think about each domain and indicate how much you generally want to make the best choice in the respective domain or whether it is enough for you to choose an option that is satisfactory and good enough. (1 = ‘Good enough’ is enough for me; 6 = I want to make the best choice)

- Smartphone
- Detergent
- Furniture
- Laptop
- Bottled water
- Clothes
- Food
- Shoes
- Sunglasses
- Perfume
- Car
- Restaurant
- Café/Bar
- Hotel room
- Holiday destination
- Film
- Book
- Meal (e.g., in a restaurant)
- Drink (e.g., in a bar)
- TV series
- Concert
- Gym
- Studies
- Job
- Employer
- Friends
- Partner
- Apartment
- Area of residence
